# Preclinical Investigations of PM01183 (Lurbinectedin) as a Single Agent or in Combination with Other Anticancer Agents for Clear Cell Carcinoma of the Ovary

**DOI:** 10.1371/journal.pone.0151050

**Published:** 2016-03-17

**Authors:** Ryoko Takahashi, Seiji Mabuchi, Mahiru Kawano, Tomoyuki Sasano, Yuri Matsumoto, Hiromasa Kuroda, Katsumi Kozasa, Kae Hashimoto, Kenjiro Sawada, Tadashi Kimura

**Affiliations:** Department of Obstetrics and Gynecology, Osaka University Graduate School of Medicine, 2–2 Yamadaoka, Suita, Osaka, 565–0871, Japan; University of Quebec at Trois-Rivieres, CANADA

## Abstract

**Objective:**

The objective of this study was to evaluate the antitumor effects of lurbinectedin as a single agent or in combination with existing anticancer agents for clear cell carcinoma (CCC) of the ovary, which is regarded as an aggressive, chemoresistant, histological subtype.

**Methods:**

Using human ovarian CCC cell lines, the antitumor effects of lurbinectedin, SN-38, doxorubicin, cisplatin, and paclitaxel as single agents were assessed using the MTS assay. Then, the antitumor effects of combination therapies involving lurbinectedin and 1 of the other 4 agents were evaluated using isobologram analysis to examine whether these combinations displayed synergistic effects. The antitumor activity of each treatment was also examined using cisplatin-resistant and paclitaxel-resistant CCC sublines. Finally, we determined the effects of mTORC1 inhibition on the antitumor activity of lurbinectedin-based chemotherapy.

**Results:**

Lurbinectedin exhibited significant antitumor activity toward chemosensitive and chemoresistant CCC cells *in vitro*. An examination of mouse CCC cell xenografts revealed that lurbinectedin significantly inhibits tumor growth. Among the tested combinations, lurbinectedin plus SN-38 resulted in a significant synergistic effect. This combination also had strong synergistic effects on both the cisplatin-resistant and paclitaxel-resistant CCC cell lines. Everolimus significantly enhanced the antitumor activity of lurbinectedin-based chemotherapies.

**Conclusions:**

Lurbinectedin, a new agent that targets active transcription, exhibits antitumor activity in CCC when used as a single agent and has synergistic antitumor effects when combined with irinotecan. Our results indicate that lurbinectedin is a promising agent for treating ovarian CCC, both as a first-line treatment and as a salvage treatment for recurrent lesions that develop after platinum-based or paclitaxel treatment.

## Introduction

Clear cell carcinoma (CCC) of the ovary is known to be less sensitive to platinum-based first-line chemotherapy and to be associated with a worse prognosis than serous adenocarcinoma (SAC), a more common histological subtype of ovarian cancer [1–4]. On the basis of previous preclinical and clinical studies suggested that irinotecan is more effective in CCC cells than other anticancer agents [5,6], a phase III study comparing the activity of irinotecan plus cisplatin versus carboplatin plus paclitaxel as a first-line treatment for CCC was conducted by the Japanese Gynecologic Oncology Group (JGOG) (protocol JGOG3017). However, this study failed to demonstrate the superiority of irinotecan plus cisplatin over carboplatin plus paclitaxel [7]. The lack of an effective chemotherapy for recurrent CCC is another important clinical problem [8]. Therefore, novel treatment strategies for CCC (for both first-line treatment and salvage treatment for recurrent disease) are required.

Trabectedin, an anticancer agent, has recently become the focus of attention for researchers investigating the treatment of CCC. On the basis of the results of a phase III clinical study (the OVA301 study) [9], the use of trabectedin in combination with pegylated liposomal doxorubicin was approved by the European Union in 2009 as a treatment for relapsed platinum-sensitive ovarian cancer. Trabectedin interacts with the nucleotide excision repair (NER) machinery, a versatile DNA repair system that acts against DNA damage induced by platinum-based agents [10], in an unusual manner. An elegant study demonstrated that NER-deficient cells (deficient in NER-related genes) exhibited resistance to trabectedin and that their sensitivity to trabectedin was restored by the transfection of the corresponding genes [11]. These findings are in clear contrast with the results obtained for platinum-based agents [12]. Thus, CCC that display increased NER activity [11], might be the candidates for trabectedin treatment. Consistent with the promising results obtained in preclinical studies of ovarian CCC [13,14], a phase II study involving recurrent ovarian CCC patients showed that combination therapy with trabectedin and temsirolimus exhibited significant activity, with a response rate of 14.3% and a clinical benefit rate of 42.9% [15].

PM01183, which is also known as lurbinectedin, is a novel synthetic agent derived from trabectedin. It is a covalent DNA minor groove binder and is structurally similar to trabectedin, although the tetrahydroisoquinoline present in trabectedin is replaced with a tetrahydro β-carboline. The latter structural difference confers pharmacokinetic and pharmacodynamic benefits, which result in decreased toxicity and enable the use of treatment regimens with increased dose intensities. Consequently, lurbinectedin exhibits increased antitumor activity compared with trabectedin [16]. In phase I studies, it was found that the maximum tolerated doses of trabectedin and PM01183 (lurbinectedin) are 1.5 mg/m^2^ [17] and 5.0 mg/m^2^ [18], respectively.

In preclinical studies, PM01183 exhibited broad antitumor activity to human cancer cell lines *in vitro* [16]. It also significantly inhibited the growth of a wide variety of human cancer xenografts in athymic mice [16]. Following the encouraging results obtained in these preclinical studies and phase I-II clinical trials [19], a phase III trial investigating the activity of lurbinectedin versus pegylated liposomal doxorubicin or topotecan is currently being conducted in recurrent ovarian cancer patients [20]. However, as most of the patients in the former clinical study displayed SAC histology [19] and the ovarian cancer cell lines used in previous preclinical studies of lurbinectedin were derived from ovarian SAC [21], the therapeutic potential of lurbinectedin to ovarian CCC remains unclear.

In the current study, we evaluated the therapeutic efficacy of lurbinectedin for both chemonaive and chemorefractory ovarian CCC cells when used as a single agent or in combination with other anticancer agents *in vitro* and *in vivo*. As the inhibition of mammalian target of rapamycin complex 1 (mTORC1) significantly enhanced the therapeutic efficacy of trabectedin-based chemotherapy in previous studies [13,14], we also investigated the benefit of adding the mTORC1 inhibitor everolimus to lurbinectedin-based combination chemotherapy in CCC.

## Materials and Methods

### Reagents and antibodies

PM01183 (lurbinectedin) was obtained from PharmaMar (Madrid, Spain). Everolimus was obtained from Novartis Pharma AG (Basel, Switzerland). Cisplatin, paclitaxel, 7-ethyl-10-hydroxycamptothecin (SN-38), and doxorubicin were purchased from Sigma (St Louis, MO). Irinotecan (7-ethyl-10-[4-(1-piperidino)-1-piperidino]carbonyloxycamptothecin, CPT-11) was obtained from Yakult Honsha Co. (Tokyo, Japan). Enhanced chemiluminescence Western blotting detection reagents were purchased from Perkin Elmer (Boston, MA, USA). Antibodies recognizing poly-(ADP-ribose) polymerase (PARP) and β-actin were obtained from Cell Signaling Technology (Beverly, MA, USA). Antibodies recognizing P-glycoprotein (P-gp) were purchased from Thermo Scientific (Waltham, MA, USA). Anti-rabbit and anti-mouse secondary antibodies were purchased from Santa Cruz Biotechnology (Dallas, TX, USA). The cell titer 96-well proliferation assay kit was obtained from Promega (Madison, WI, USA).

### Drug preparation

For the in vitro analyses, lurbinectedin was prepared as a 1 μmol/L stock solution in dimethyl sulfoxide (DMSO). Everolimus was prepared in DMSO before being added to the cell cultures, as described previously [22]. Cisplatin and doxorubicin were dissolved in sterilized double–distilled water to final concentrations of 10 and 1 mmol/L, respectively. SN-38 and paclitaxel were dissolved in DMSO to final concentrations of 10 mmol/L and 100 μmol/L, respectively. For the in vivo analyses, lurbinectedin and CPT-11 were diluted to the appropriate concentration in double-distilled water just before their administration in an intravenous infusion.

### Cell culture

The human ovarian CCC cell lines RMG1, RMG2, KOC7C, and HAC2 were kindly provided by Dr. H. Itamochi (Tottori University, Tottori, Japan). These cell lines were extensively characterized in previous studies [23–26]. We authenticated these cell lines in our laboratory based on morphological observations. No further cell line authentication was conducted by the authors. The CCC cell lines were maintained as monolayer cultures in Dulbecco’s modified Eagle’s medium (DMEM Ham’s F-12; Nacalai Tesque, Kyoto, Japan) supplemented with 10% fetal bovine serum (FBS), as described previously [27]. The human ovarian SAC cell lines A2780, HeyA8, and SKOV-3 were purchased from the American Type Culture Collection (ATCC, VA, USA). The SAC cell lines were maintained as monolayer cultures in DMEM (Nacalai Tesque, Kyoto, Japan) supplemented with 10% FBS. All cell lines were maintained in a humidified incubator at 37°C in 5% CO2.

### Cell proliferation assay

The MTS assay was used to analyze the effects of each drug. Cells were plated in 96-well plates and exposed to the drugs at different concentrations. After 48 hours’ incubation, the number of surviving cells was assessed by determining the A_490nm_ of the dissolved formazan product after the addition of MTS for 1 hour, as described by the manufacturer (Promega). Cell viability was calculated as follows: A_exp group_ / A_control_ × 100. The experiments were repeated at least three times, and representative results are shown.

### Cell cycle analysis

CCC cells (2×10^5^) were incubated with lurbinectedin at the indicated concentrations for 48 hours. The cells were then fixed with 75% ethanol overnight at -20°C and stained with propidium iodide (PI; 50 μg/mL) in the presence of RNase A (100 μg/mL; Roth) for 60 minutes at 4°C. In each experiment, the cell cycle distribution was determined by analyzing 10,000 cells using a FACScan flow cytometer and Cell Quest software (Becton Dickinson, NJ, USA), as reported previously [27]. The experiments were repeated at least three times, and representative results are shown.

### Detection of apoptosis

CCC cells (2~5×10^5^) were treated with lurbinectedin, SN38 or in combination of the two drugs at the indicated concentrations for 48 hours. Then, the cells were harvested and stained with PI and annexin V using the annexin V-fluorescein isothiocyanate (FITC) apoptosis detection kit (BioVision, CA, USA), according to the manufacturer’s instructions. Fluorescence data were collected using flow cytometry, as reported previously [28]. The sum total of early apoptotic cells (annexin V(+), PI(−) cells) and late apoptotic cells (annexin V(+), PI(+) cells) was defined as the total number of apoptotic cells. The experiments were repeated at least three times, and representative results are shown.

### Isobologram method and the combination index

The isobologram method relies on determining the combined concentrations of D1 (lurbinectedin) and D2 (the drug used in combination with lurbinectedin) that result in a fractional kill value of 50%. For each experimental concentration of lurbinectedin, the concentration of D2 that would cause the desired effect when used in combination with lurbinectedin was found by non-linear fitting of the concentration-effect relationship of D2 to the given lurbinectedin concentration. Conversely, for each experimental concentration of D2, the lurbinectedin concentration that would cause the desired effect when used in combination with D2 was found by non-linear fitting of the concentration-effect relationship of lurbinectedin to the particular D2 concentration. In this manner, multiple pairs of drug concentrations that achieved the desired isoeffect were found. For each pair of drug concentrations (D_Lurbinectedin_, D_D2_) that produced a fractional kill value of 50%, the combination index (CI) was calculated as follows: CI = D_Lurbinectedin_ / IC_50Lurbinectedin_ + D_D2_ / IC_50D2_. CI values of <1 indicate synergism, CI values of 1 indicate an additive effect, and CI values of >1 indicate antagonism. The significance of the differences between the mean CI values for each combination and 1 was evaluated using a 2-tailed *t* test. The experiments were repeated at least three times, and representative results are shown.

### Western blot analysis

CCC cells were treated with lurbinectedin or other agents for appropriate periods of time, washed twice with ice cold phosphate-buffered saline (PBS), and lysed in radioimmunoprecipitation assay (RIPA) lysis buffer. The protein concentrations of the cell lysates were determined using the Bio-Rad protein assay reagent. Equal amounts of protein were applied to 5–20% polyacrylamide gels, and then the electrophoresed proteins were transblotted onto nitrocellulose membranes. After the membranes had been blocked, they were incubated with anti-PARP, anti-cleaved caspase 3, anti-P-gp, or anti-β-actin antibodies. The immunoblots were visualized with horseradish peroxidase-coupled goat anti-rabbit or anti-mouse immunoglobulins, using the enhanced chemiluminescence Western blotting system (Perkin Elmer, MA, USA).

### Subcutaneous xenograft model

All procedures involving animals and their care were approved by the animal care and usage committee of Osaka University (Osaka, Japan), in accordance with the relevant institutional and National Institutes of Health guidelines. Preliminary experiments were conducted to examine the effects of lurbinectedin on ovarian CCC. Five- to 7-week-old nude mice (n = 12) had 1×10^7^ RMG1 cells in 150 μL of PBS s.c. injected into their left flanks. When the tumors reached about 50 mm^3^ in size, the mice were assigned to one of two treatment groups. The first group (n = 6) was i.v. administered PBS, and the second group (n = 6) was i.v. administered lurbinectedin (0.180 mg/kg) each week for 6 weeks. The dose of lurbinectedin (0.180 mg/kg) used was based on that employed in a previous preclinical study of ovarian cancer, in which it showed significant *in vivo* antitumor activity [21]. A second set of experiments was conducted to examine the antitumor effects of combination treatment involving lurbinectedin and irinotecan. We employed irinotecan in the *in vivo* experiments because the clinical use of SN-38 is limited by its poor aqueous solubility [29], and the goal of this study was to identify practical treatments that could be used in the clinical setting. Five- to 7-week-old nude mice (n = 18) had 1×10^7^ RMG1 cells in 150 μL of PBS s.c. injected into their flanks. When the tumors reached about 50 mm^3^ in size, the mice were assigned to 1 of 3 treatment groups, which received PBS, CPT-11 (50 mg/kg weekly), or lurbinectedin (0.180 mg/kg weekly) plus CPT-11 (50 mg/kg weekly). Caliper measurements of the longest perpendicular diameter of each tumor were obtained twice a week and used to estimate tumor volume according to the following formula: *V = L × W × D × π / 6*, where *V* is the volume, *L* is the length, *W* is the width, and *D* is the depth.

### Establishment of chemoresistant cell lines

Lurbinectedin-resistant sublines derived from RMG1 cells were developed in our laboratory by continuously exposing the cells to lurbinectedin. Briefly, RMG1 cells were exposed to stepwise increases in the concentration of lurbinectedin. The cells were initially exposed to a lurbinectedin concentration of 0.1 nmol/L. After the cells had regained their exponential growth rate, the lurbinectedin concentration was increased by 0.1–0.3 nmol/L, and the procedure was repeated until the concentration was >5.0 nmol/L.

Cisplatin-resistant CCC sublines (RMG1-CR and RMG2-CR) and paclitaxel-resistant CCC sublines (RMG1-PR and RMG2-PR) derived from CCC cells (RMG1 and RMG2) were also developed by continuously exposing CCC cells to cisplatin and paclitaxel, as reported previously [13,22].

### RNA interference

siRNA that specifically targeted multidrug resistance protein 1 (MDR-1, also known as P-gp) and a non-targeting control siRNA were purchased from Santa Cruz Biotechnology (Santa Cruz, CA, USA). Lurbinectedin-resistant RMG1 cells (RMG1-LR) were transfected with siRNA using Lipofectamine 2000 (Invitrogen, Carlsbad, CA, USA).

### Statistical analysis

Statistical analysis was done by using Wilcoxon’s exact test, or one-way ANOVA with Bonferroni’s method where appropriate. *P*-values of <0.05 were considered significant.

## Results

### In vitro growth-inhibitory effects of lurbinectedin on CCC cell lines

**Fig 1:**
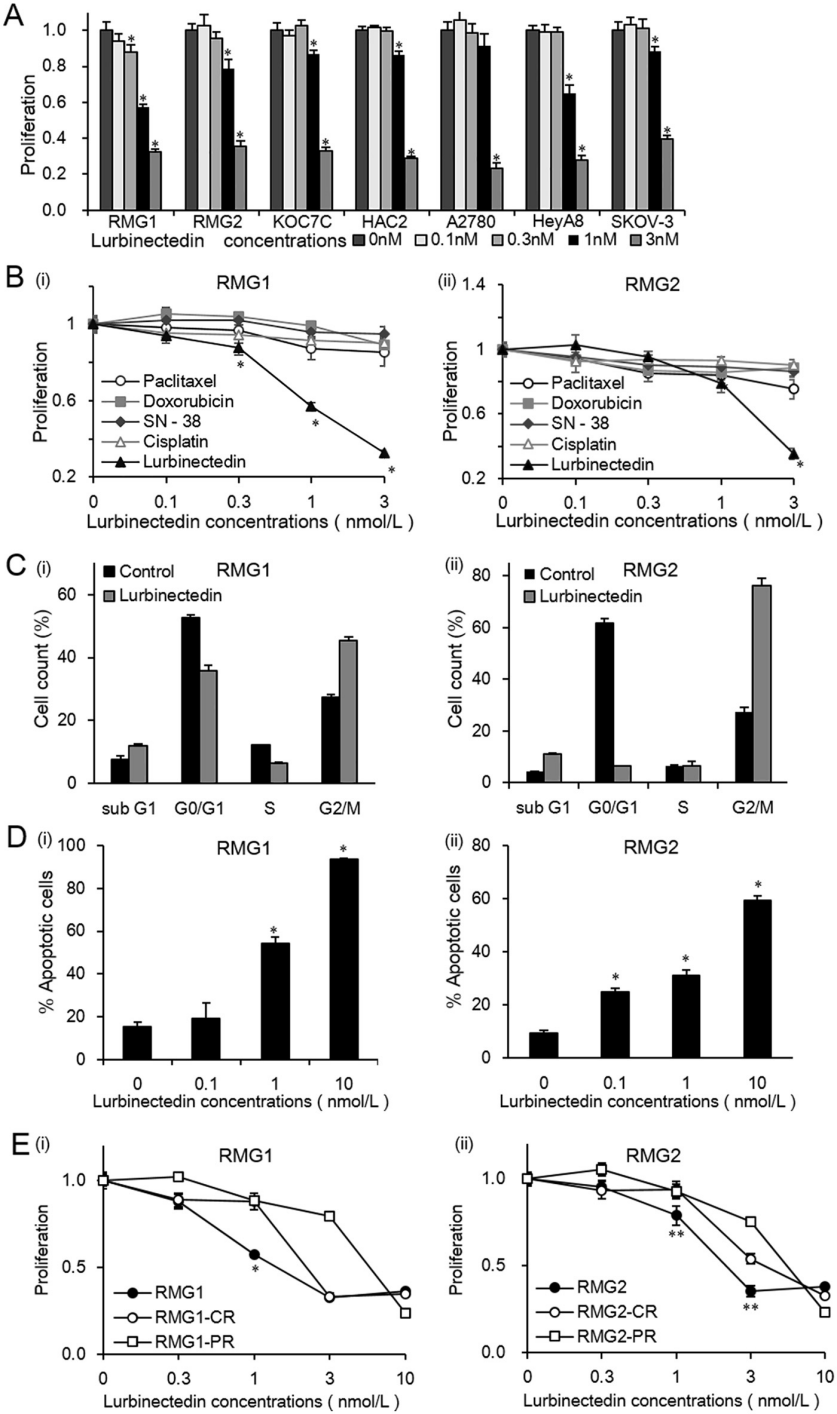
*In vitro* growth-inhibitory effects of lurbinectedin as a single agent. A, Sensitivity of CCC and SAC cells to lurbinectedin. CCC (RMG1, RMG2, KOC7C, and HAC2) cells and SAC (A2780, HeyA8, and SKOV-3) cells were treated with the indicated concentrations of lurbinectedin in the presence of 10% FBS for 48 hours. Cell proliferation was assessed using the MTS assay. Data points, mean values; bars, SD (*, significantly different from the control; P <0.05). B, Comparison of the growth-inhibitory activities of 5 anticancer agents. CCC (RMG1 and RMG2) cells were treated with the indicated concentrations of paclitaxel, doxorubicin, SN-38, cisplatin, or lurbinectedin in the presence of 10% FBS for 48 hours. Cell proliferation was assessed using the MTS assay. Data points, mean values; bars, SD (*, significantly different from paclitaxel; P <0.05). C, Lurbinectedin alters the cell cycle distribution of CCC cells. CCC cells (RMG1 and RMG2) were treated with lurbinectedin at a concentration of 1 nmol/L for 48 hours. Then, the cells were detached and fixed with 75% ethanol at -20°C overnight. The cells were subsequently incubated with 100 μg/ml RNase A and 50 μg/ml PI in the dark. A total of 1×10^4^ cells were subjected to flow cytometry. The bar graphs show the percentages of RMG1 and RMG2 cells that had been treated (or not) with lurbinectedin in the subG1, G0/G1, S, and G2/M phases. Data points, mean values; bars, SD. D, Lurbinectedin induces apoptosis in CCC cells. CCC cells (RMG1 and RMG2) were treated with lurbinectedin at concentrations of 0, 0.1, 1, and 10 nmol/L for 48 hours. Then, the cells were detached and subjected to dual staining with both annexin V and FITC, and cellular DNA was stained using PI. A total of 1×10^4^ cells were subjected to flow cytometric analysis. Data points, mean values; bars, SD (*, significantly different from the control; P <0.05). E, Effects of lurbinectedin on the growth of chemoresistant CCC cells. Cisplatin- and paclitaxel-resistant sublines were established as described in the Materials and Methods section. The parental (RMG1 and RMG2), cisplatin-resistant (RMG1-CR and RMG2-CR), and paclitaxel-resistant (RMG1-PR and RMG2-PR) cells were treated with the indicated concentrations of lurbinectedin in the presence of 10% FBS for 48 hours. Cell proliferation was assessed using the MTS assay. Data points, mean values; bars, SD (*, significantly different from the RMG1-CR or RMG1-PR cells; **, significantly different from the RMG2-CR or RMG2-PR cells; P <0.05); CR, cisplatin resistant; PR, paclitaxel resistant. All experiments were repeated at least three times, producing similar results, and representative results are shown.

To examine the effects of lurbinectedin on the proliferation of ovarian cancer cells of CCC origin, we conducted MTS assays using 4 human ovarian cancer cell lines. As shown in Fig 1A, 48 hours’ treatment with lurbinectedin inhibited the proliferation of the cell lines in a dose-dependent manner. The antiproliferative effects of lurbinectedin on the CCC cells were similar to its effects on SAC cells (Fig 1A). Using the RMG1 and RMG2 cell lines, we next compared the antitumor effects of lurbinectedin with those of paclitaxel; doxorubicin; SN-38, which is an active metabolite of irinotecan; and cisplatin (Fig 1B). The IC_50_ values obtained in each experiment are summarized in Table 1. Lurbinectedin demonstrated significantly greater antitumor activity in the CCC cell lines than the other anticancer agents.

**Table 1:** IC_50_ values of anticancer drugs in human CCC cell lines.

		IC_50_	
Drug		RMG1	RMG2
Cisplatin	μM	20.56 (1.36)	35.26 (7.94)
SN-38	μM	0.27 (0.09)	0.31 (0.09)
Doxorubicin	nM	75.21 (13.11)	55.72 (12.42)
Paclitaxel	nM	17.13 (0.54)	5.79 (1.61)
Lurbinectedin	nM	1.25 (0.08)	1.16 (0.09)

The IC_50_ values represent the mean of at least 3 independent experiments (SD)

### The mechanisms responsible for the antiproliferative effects of lurbinectedin

We examined the effects of lurbinectedin on cell cycle progression and apoptosis. As shown in Fig 1C, the percentage of cells in the G2/M phase was significantly increased by 2 days’ treatment with lurbinectedin. In both cell lines, the percentage of apoptotic cells in the sub-G1 peak was also increased after treatment with lurbinectedin. Moreover, as shown in Fig 1D, the treatment of CCC cells with lurbinectedin induced apoptosis in a dose-dependent manner.

### Effects of trabectedin on cisplatin- or paclitaxel-resistant CCC in vitro

We next examined the growth-inhibitory effects of lurbinectedin on chemoresistant CCC cells. For this purpose, we employed cisplatin-resistant and paclitaxel-resistant CCC cells, as well as their respective parental cell lines. As shown in Fig 1E, treatment with lurbinectedin inhibited the proliferation of all of these CCC cells in a dose-dependent manner. The antitumor effects of lurbinectedin on the cisplatin- and paclitaxel-resistant CCC cells were slightly milder than those observed in their respective parental cell lines at lower concentrations (0.3–3 nmol/L). However, at a concentration of 10 nmol/L the antitumor effects of lurbinectedin on the cisplatin- and paclitaxel-resistant CCC cells were equivalent to those seen in their respective parental cells.

### In vivo growth-inhibitory effects of lurbinectedin on ovarian CCC

**Fig 2:**
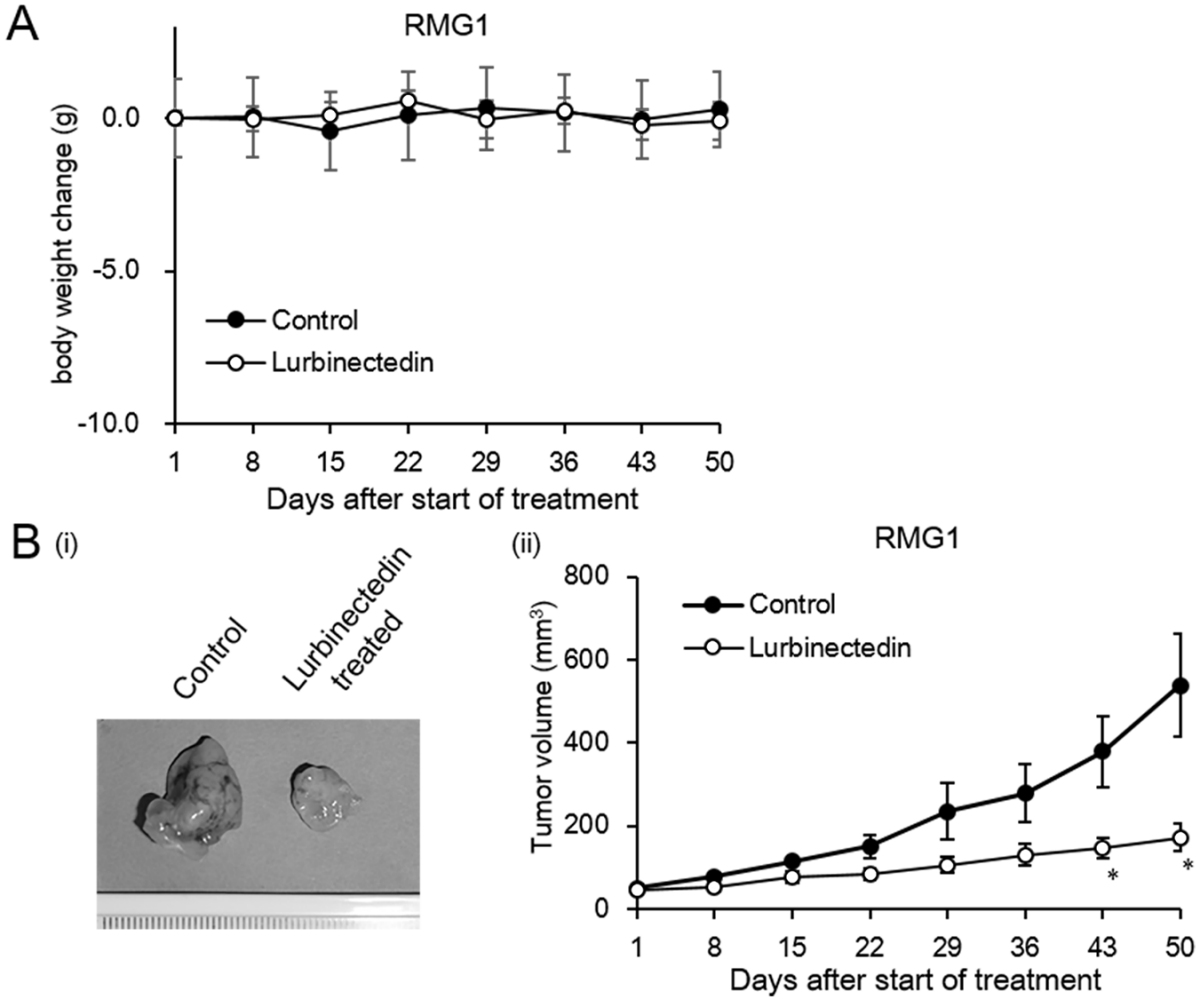
*In vivo* growth-inhibitory effects of lurbinectedin as a single agent. Athymic nude mice were s.c. inoculated with RMG1 cells. At 2–3 weeks after the inoculation procedure, the mice were i.v. administered PBS or 0.180 mg/kg lurbinectedin each week for 6 weeks. A, Graphs depicting the weekly changes in mouse body weight seen in each treatment group. Data points, mean values; bars, SD (*, significantly different from the control; P <0.05). B, Appearance of s.c. tumors and graphs depicting the weekly changes in tumor volume (mm^3^) observed in each treatment group. Data points, mean values; bars, SD (*, significantly different from the control; P <0.05).

To examine the in vivo growth-inhibitory effects of lurbinectedin, we employed an s.c. xenograft model in which athymic mice were s.c. inoculated with RMG1 cells. Overall, the drug treatment was well tolerated throughout the study and did not cause any apparent toxicities. The changes in the body weights of the mice are shown in Fig 2A. As shown in Fig 2B, the mean RMG1-derived tumor burden in the mice treated with lurbinectedin was 171.9 mm^3^, whereas it was 537.3 mm^3^ in the PBS-treated mice. Overall, treatment with lurbinectedin decreased the RMG1-derived tumor burden by 68.0% compared with PBS treatment.

### Effects of combination treatment with lurbinectedin and other antineoplastic agents

**Fig 3:**
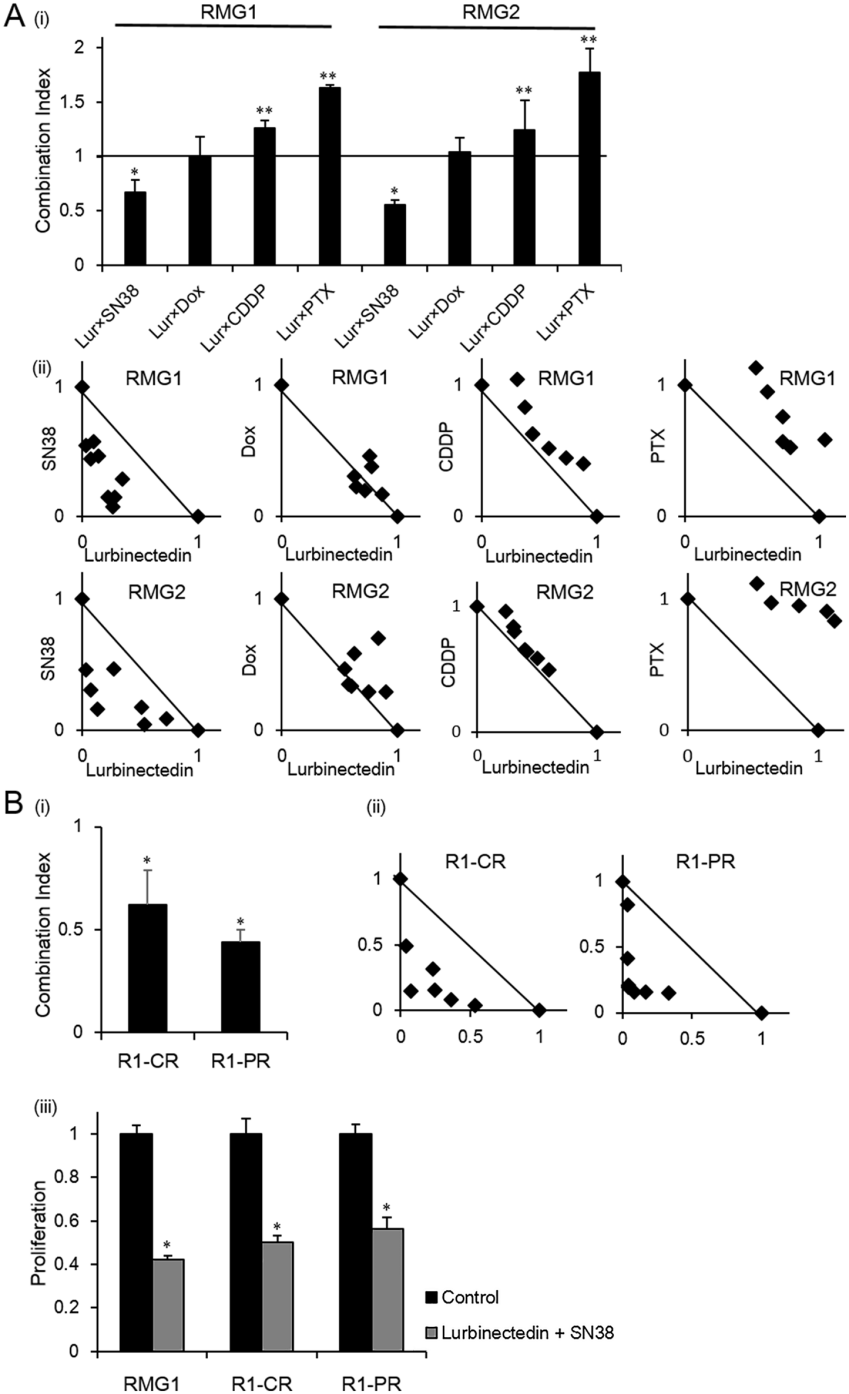
*In vitro* growth-inhibitory effects of combination treatments involving lurbinectedin. A, (i) Combination Index of the combination treatment involving lurbinectedin. CCC cell lines (RMG1 and RMG2) were treated with a combination of lurbinectedin and 1 of 4 agents (SN-38, doxorubicin, cisplatin, or paclitaxel) in the presence of 10% FBS for 48 hours. Cell viability was assessed using the MTS assay. The CI for each treatment was calculated as described in the Materials and Methods section. Data are shown as mean values derived from at least 3 independent experiments; bars, SD (*, significantly lower than 1; **, significantly higher than 1; P <0.05). (ii) Representative isobologram of the treatment of RMG1 and RMG2 cells with combination treatment involving lurbinectedin. All experiments were repeated at least three times, producing similar results, and representative results are shown. B, (i) Combination Index of combination treatment involving lurbinectedin and SN-38 on the survival of cisplatin- and paclitaxel-resistant CCC cells. Cisplatin-resistant cells (RMG1-CR) and paclitaxel-resistant cells (RMG1-PR) were treated with a combination of lurbinectedin and SN-38 in the presence of 10% FBS for 48 hours. Cell viability was assessed using the MTS assay. The CI for each treatment was calculated as described in the Materials and Methods section. Data are shown as mean values derived from at least 3 independent experiments; bars, SD (*, significantly lower than 1; P <0.05). (ii) Representative isobologram of the treatment of RMG1-CR and RMG1-PR cells with a combination of lurbinectedin and SN-38. (iii) Effects of combination treatment involving lurbinectedin and SN-38 on the survival of cisplatin- and paclitaxel-resistant CCC cells. RMG1 cells, cisplatin-resistant CCC cells (RMG1-CR), and paclitaxel-resistant CCC cells (RMG1-PR) were treated with lurbinectedin (0.3 nM) and SN-38 (60 nM) in the presence of 10% FBS for 48 hours. Cell viability was assessed using the MTS assay. Data points, mean values; bars, SD (*, significantly different from the control; P <0.05). CDDP, cisplatin; DOX, doxorubicin; PTX, paclitaxel. All experiments were repeated three times, producing similar results, and representative results are shown.

We next investigated the chemotherapeutic agents that have the strongest synergistic effects in ovarian CCC when combined with lurbinectedin. Fig 3A(i) shows the CI values for each combination treatment (lurbinectedin plus SN-38, doxorubicin, cisplatin, or paclitaxel) obtained using the RMG1 and RMG2 cell lines. Representative isobolograms are also shown in Fig 3A(ii). Among the 4 combination treatments, SN-38 combined with lurbinectedin demonstrated the lowest CI values in both the RMG1 (0.67) and RMG2 (0.56) cells, which indicated that this combination exhibited the strongest synergism in both cell lines. Combination treatment with lurbinectedin and doxorubicin demonstrated CI values of roughly 0.8 to 1.2 in the RMG1 cells and 0.8 to 1.1 in the RMG2 cells, indicating that it had additive effects. The combination treatments involving cisplatin or paclitaxel exhibited CI values of higher than 1, indicating that they had antagonistic effects. Moreover, treatment with lurbinectedin plus SN-38 displayed strong synergistic effects (Fig 3B(i) and 3B(ii)), which resulted in significant antiproliferative effects (Fig 3B(iii)) in the chemoresistant CCC cell lines.

**Fig 4:**
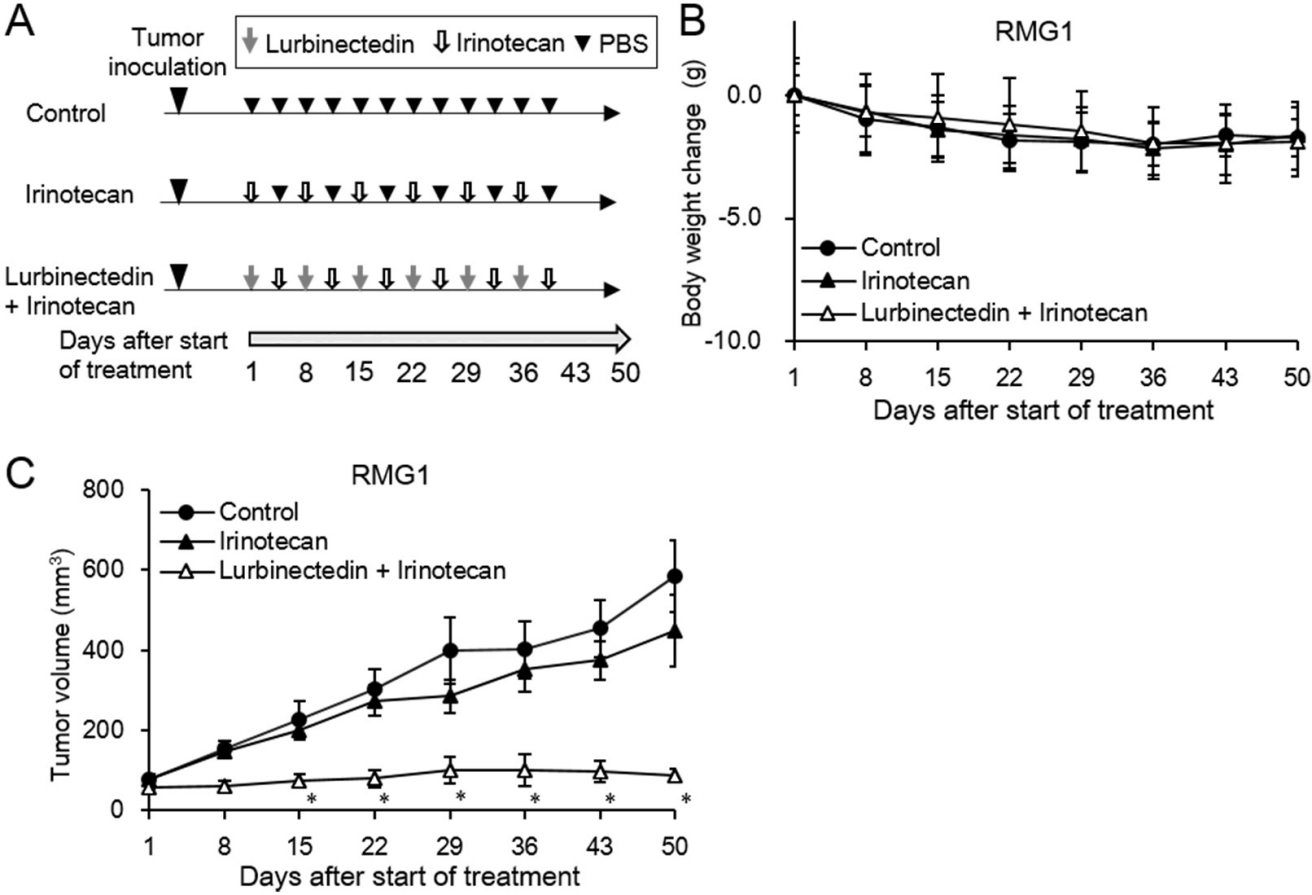
*In vivo* growth-inhibitory effects of combination treatment involving lurbinectedin and irinotecan. Athymic nude mice were s.c. inoculated with RMG1 cells. At 2–3 weeks after the inoculation procedure, the mice were i.v. administered PBS, 50 mg/kg irinotecan, or 0.180 mg/kg lurbinectedin plus 50 mg/kg irinotecan each week for 6 weeks. A, Schematic regimen for the combination treatment. B, Graphs depicting the weekly mouse body weight changes observed in each treatment group. Data points, mean values; bars, SD (*, significantly different from the control; P <0.05). C, Graphs depicting the weekly changes in tumor volume (mm^3^) in each treatment group Data points, mean values; bars, SD (*, significantly different from the control; P <0.05).

We then examined the in vivo growth-inhibitory effects of combination treatment with lurbinectedin and irinotecan (Fig 4A). Overall, the drug treatment was well tolerated throughout the study and did not cause any apparent toxicities. The changes in the animals’ body weights are shown in Fig 4B. Treatment with lurbinectedin in combination with irinotecan decreased the RMG1-derived tumor burden by 85.1% compared with PBS treatment (Fig 4C). Importantly, the growth-inhibitory effects of lurbinectedin plus irinotecan were significantly stronger than those of single-agent irinotecan or lurbinectedin (Fig 2B).

### The mechanisms responsible for the synergism between lurbinectedin and irinotecan

**Fig 5:**
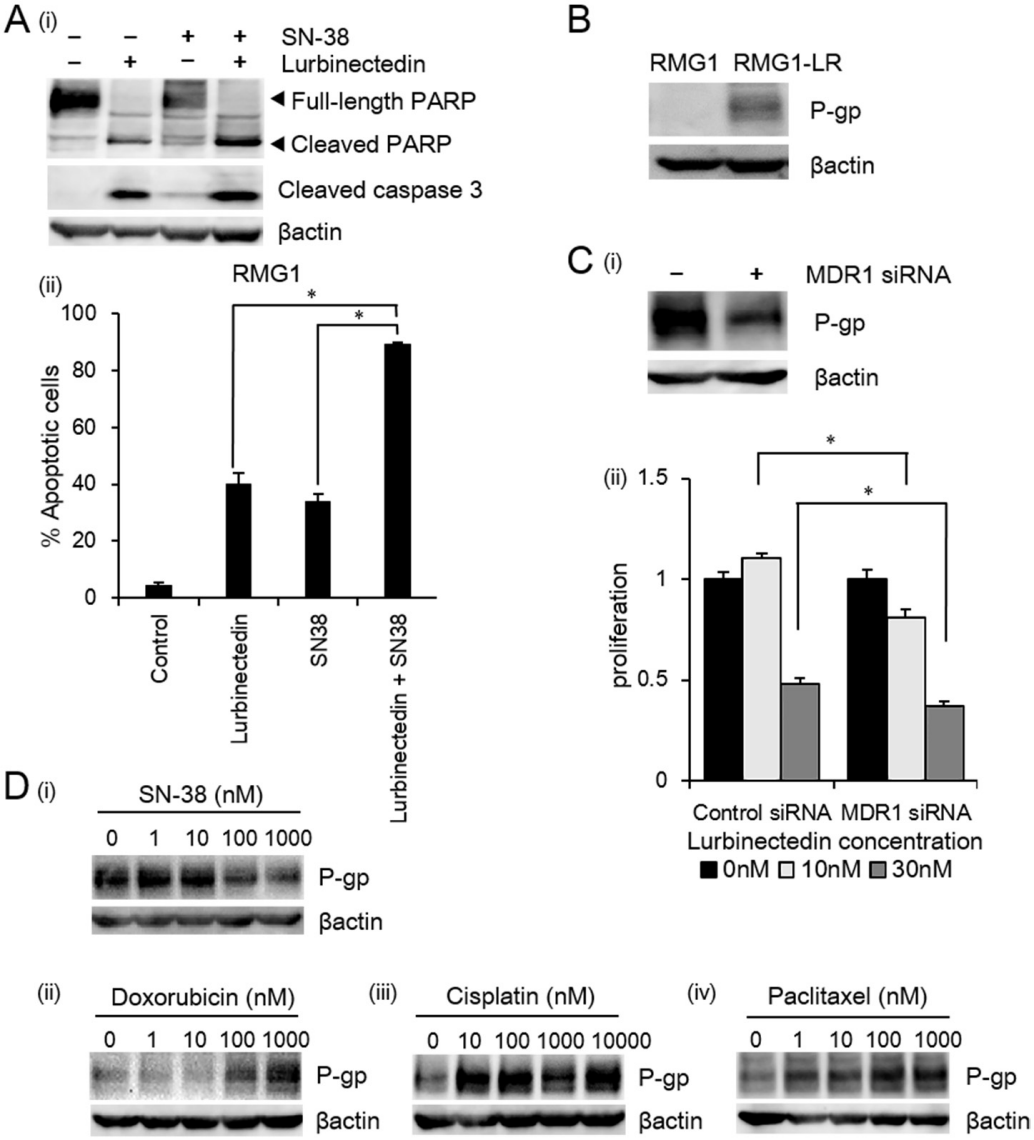
The mechanisms responsible for the synergism observed between lurbinectedin and irinotecan. A, Effects of combination treatment with lurbinectedin and SN-38 on the induction of apoptosis. (i) RMG1 cells were treated with lurbinectedin (3nM) and SN-38 (1μM) for 48 hours. The resultant cell lysates were subjected to sodium dodecyl sulfate polyacrylamide gel electrophoresis (SDS-PAGE), followed by Western blotting with anti-PARP, anti-cleaved caspase-3 and anti-β-actin antibodies. (ii) RMG1 cells were treated with lurbinectedin (0.5nM) and SN-38 (0.2μM) for 48 hours. The cells were detached and subjected to dual staining with both annexin V and FITC, and cellular DNA was stained using PI. A total of 1×10^4^ cells were subjected to flow cytometric analysis. Data points, mean values; bars, SD (*, significantly different; P <0.05). B, Expression of P-gp in lurbinectedin-sensitive (RMG1) and lurbinectedin-resistant RMG1 (RMG1-LR) cells. C, The effects of MDR-1 inhibition on the antitumor activity of lurbinectedin. (i) Suppression of MDR-1 by siRNA. Lurbinectedin-resistant RMG1 cells (RMG1-LR) were plated in 6-well plates and then transfected with 33 nM of the control or MDR-1 siRNA. Forty-eight hours after the transfection procedure, the cells were harvested, and their MDR-1 expression was assessed by Western blotting. (ii) Forty-eight hours after the transfection procedure, the cells were plated in 96-well plates and then were treated with 10nM and 30 nM of lurbinectedin for 48 hours in the presence of 10% FBS. Cell viability was assessed using the MTS assay. Data points, mean values; bars, SD (*, significantly different from the control; P <0.05). D, Effects of anti-cancer agents on the expression of P-gp. RMG1-LR cells were treated with the indicated concentrations of SN-38, doxorubicin, cisplatin or paclitaxel for 48 hours. The resultant cell lysates were subjected to SDS-PAGE, followed by Western blotting with anti-P-gp and anti-β-actin antibodies. All experiments were repeated at least three times, producing similar results, and representative results are shown.

We first examined the effects of SN-38 on lurbinectedin-induced apoptosis in CCC cells. As shown in Fig 5A(i), the addition of SN-38 to lurbinectedin enhanced the lurbinectedin-induced cleavage of both PARP and Caspase-3 in the RMG1 cells. Moreover, as shown in Fig 5A(ii), co-treatment with SN-38 and lurbinectedin induced apoptosis in a significantly greater number of cells than lurbinectedin treatment alone, indicating that SN-38 enhanced the antitumor activity of lurbinectedin by promoting apoptosis. A similar result was obtained for the RMG2 cells (data not shown).

We next investigated whether P-gp, a member of the ATP-binding cassette transporter superfamily, was involved in the observed synergistic effects. For this purpose, we established lurbinectedin-resistant sublines (RMG1-LR) derived from RMG1 cells. As shown in Fig 5B, significantly greater P-gp expression was observed in the lurbinectedin-resistant cell line than in the parental cell line. Moreover, the sensitivity of the RMG1 cells to lurbinectedin was found to be significantly associated with the extent of their P-gp expression (Fig 5B and 5C). Collectively, these results indicate that P-gp is involved in lurbinectedin resistance. Treatment with single-agent SN-38 significantly reduced the expression of P-gp in a dose-dependent manner (Fig 5D(i)); however, no such effects were observed in the cells treated with doxorubicin, cisplatin or paclitaxel (Fig 5D(ii)–5D(iv)).

### mTOR inhibition enhances the antitumor activity of lurbinectedin-based chemotherapy

**Fig 6:**
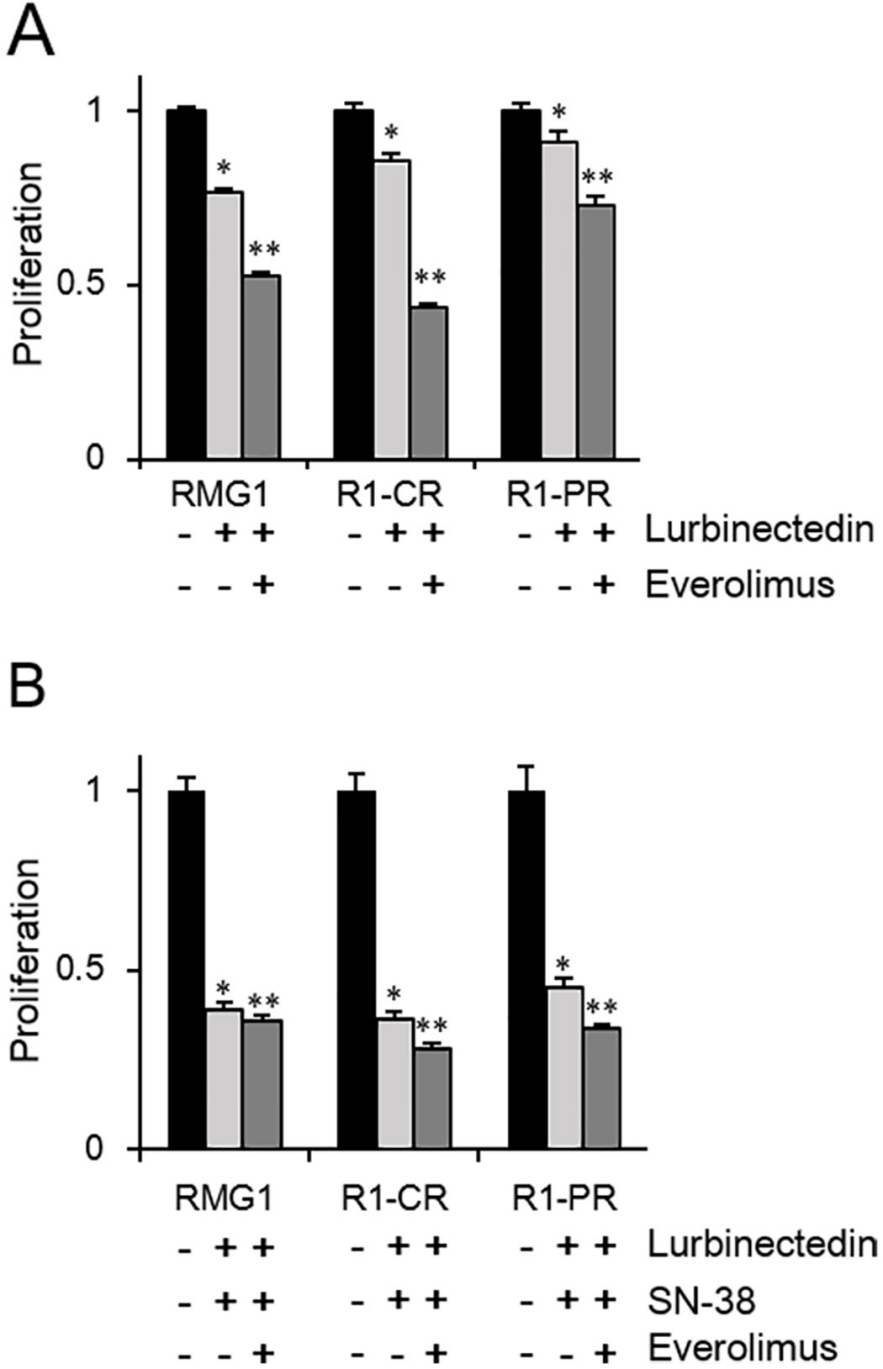
The effects of mTORC1 inhibition on the activity of lurbinectedin. A, Effects of the addition of everolimus treatment to lurbinectedin. RMG1 cells, cisplatin-resistant CCC cells (RMG1-CR), and paclitaxel-resistant CCC cells (RMG1-PR) were treated with lurbinectedin (0.7 nM) with or without everolimus (100 nM) in the presence of 10% FBS for 48 hours. Cell viability was assessed using the MTS assay. Data points, mean values; bars, SD (*, significantly different from the control; **, significantly different from lurbinectedin; P <0.05). B, Effects of the addition of everolimus to combination treatment involving lurbinectedin and SN-38. RMG1 cells, cisplatin-resistant CCC cells (RMG1-CR), and paclitaxel-resistant CCC cells (RMG1-PR) were treated with lurbinectedin (0.5 nM) plus SN-38 (100 nM) with or without everolimus (100 nM) in the presence of 10% FBS for 48 hours. Cell viability was assessed using the MTS assay. Data points, mean values; bars, SD (*, significantly different from the control; **, significantly different from SN-38 plus lurbinectedin; P <0.05). All experiments were repeated three times, producing similar results, and representative results are shown.

We examined whether the addition of everolimus would enhance the antitumor activity of lurbinectedin-based chemotherapy. Treatment with everolimus significantly enhanced the antitumor effects of lurbinectedin (Fig 6A) and lurbinectedin plus SN-38 in both the parental and chemoresistant CCC cell lines (Fig 6B).

## Discussion

In the current study, we have shown that the in vitro growth-inhibitory effects of lurbinectedin to CCC of the ovary were stronger than those of other existing anticancer agents (Fig 1B). We also found that treating mice with lurbinectedin significantly inhibited the growth of CCC-derived tumors without causing any apparent toxicities. These findings indicate that lurbinectedin exhibits significant clinical activity as a single agent for CCC in the setting of first-line therapy.

It is unclear why CCC cells are highly sensitive to lurbinectedin. However, recent preclinical investigations have suggested that the damage caused to tumor cells by lurbinectedin is not subject to NER repair [30], and this might explain why CCC cells, which exhibit cisplatin-resistance due to their increased NER activity, are sensitive to lurbinectedin.

We also found that combination treatment with lurbinectedin and SN-38 produced the strongest synergistic effect in CCC cells in vitro, which agrees with our previous finding that combination treatment with trabectedin and SN-38 resulted in synergistic effects in CCC cells [14]. According to previous clinical studies, the peak plasma concentrations of SN-38 and lurbinectedin are 166.0 nmol/L and 351.7nmol/L, respectively, [18,31,32] indicating that the lurbinectedin-based chemotherapies proposed in the current study are reasonable and clinically achievable, as the concentrations of SN-38 and lurbinectedin employed in the current in vitro experiments were lower than the abovementioned values.

The mechanisms underlying the synergistic effects of combination treatment with lurbinectedin and SN-38 remain unknown. Irinotecan is an inhibitor of the type I topoisomerase enzyme, which binds to topoisomerase I DNA complex, resulting in the formation of irreversible double-strand breaks, and hence, cell death [33]. In contrast, lurbinectedin binds covalently to the DNA minor groove, forming adducts that can induce double-strand breaks, which leads to the inhibition of transcription. The accumulation of DNA damage delays cell cycle progression and ultimately triggers apoptotic cell death [34]. Thus, combining lurbinectedin with SN-38 might result in increased genotoxicity in CCC cells due to the induction of multiple DNA damage mechanisms.

Although a previous preclinical study of ovarian cancer involving an SAC line suggested that lurbinectedin and cisplatin act synergistically in vivo [21], in the current study lurbinectedin and cisplatin did not have synergistic effects to the CCC cell lines. Combination treatment with lurbinectedin and cisplatin, doxorubicin or paclitaxel exhibited additive and antagonistic effects (Fig 3A), respectively, which was in clear contrast with the results obtained for the combination of lurbinectedin plus SN-38. The mechanism responsible for the differential responses of these combinations remains unknown. To investigate this, we examined the role of P-gp, which is encoded by the MDR-1 gene and is known to be involved in multidrug resistance, including resistance to trabectedin [35]. As shown in Fig 5B and 5C, we found that P-gp is involved in lurbinectedin resistance and that treatment with SN-38 significantly attenuates the expression of P-gp in lurbinectedin-resistant CCC cells (Fig 5D). In contrast, treatment with cisplatin, doxorubicin or paclitaxel resulted in a slight increase in P-gp expression. These results indicate that P-gp expression might be, at least in part, responsible for the differential treatment responses observed in the current study (Fig 3A) and that the reduction in P-gp expression induced by SN-38 is involved in the observed synergistic effects of lurbinectedin and SN-38 in CCC cells. The sensitivity of cancer cells to chemotherapeutic agents is regulated by various mechanisms including pro- and anti-apoptotic signals, drug efflux pump activity, and/or nuclear excision repair pathways. It was recently reported that an autophagy inhibitor enhanced the antitumor effects of cisplatin in ovarian cancer [36]. Thus, these mechanisms might have affected the efficacy of lurbinectedin-based combination chemotherapies in our experimental model. Further studies are needed to obtain a deeper understanding of the mechanisms underlying the additive, synergistic, or antagonistic effects of lurbinectedin-based combination chemotherapy.

Another important finding of our study was that single-agent lurbinectedin and lurbinectedin plus SN-38 both displayed significant activity in cisplatin-resistant and paclitaxel-resistant CCC. This result is not surprising as a previous study obtained similar findings regarding the effects of trabectedin on chemoresistant CCC cells [13,14]. However, this is clinically very important because the lack of an effective chemotherapy for recurrent CCC that develops after first-line platinum-based combination chemotherapy is a major clinical problem in the management of CCC. As the RMG1-CR, RMG1-PR, RMG2-CR, and RMG2-PR cells used in this study mimic the clinical resistance seen in cisplatin-treated or paclitaxel-treated patients, our results suggest that cisplatin and paclitaxel refractory CCC are also candidates for lurbinectedin-based chemotherapy.

Importantly, as shown in Fig 6A, treatment with everolimus, an mTORC1 inhibitor, enhanced the antitumor effects of combination treatment involving lurbinectedin chemotherapy in our experimental model. Based on the findings of a preclinical study that showed that mTORC1 is frequently activated in ovarian CCC [22], the Gynecologic Oncology Group is currently conducting a phase II trial (protocol GOG0268) examining the efficacy of combination treatment with temsirolimus and carboplatin plus paclitaxel as a first-line chemotherapy for patients with stage III to IV CCC of the ovary [37]. The clinical activity induced by combined treatment involving lurbinectedin-based chemotherapy and an mTORC1 inhibitor should also be investigated in future trials in patients with ovarian CCC.

We have to recognize the potential weakness of our experimental design; i.e., we used a subcutaneously inoculated xenograft model. As our mouse model involved a heterotopic implantation site and peritoneal dissemination is the main process responsible for the progression of human ovarian cancer, further studies involving an intraperitoneal model or a genetically engineered mouse model of ovarian cancer would be useful.

In conclusion, we have shown that lurbinectedin, a new agent that targets active transcription, exhibits antitumor activity in CCC when used as a single agent and that it has synergistic effects in CCC when combined with irinotecan. Our results indicate that lurbinectedin is a promising agent for treating ovarian CCC, both as a first-line treatment and as a salvage treatment for recurrent lesions that develop after platinum-based or paclitaxel treatment. We consider that our preclinical data provide significant scientific support for future clinical trials of lurbinectedin in this patient population.
